# Female genital mutilation/cutting burden and perceptions among asylum-seeking women in Berlin, Germany: A cross-sectional study

**DOI:** 10.1016/j.jmh.2026.100416

**Published:** 2026-05-15

**Authors:** M.H. Barghouth, E. Kusi, A. Mueller, M. Kern, N. Bethke, J. Seybold, S. Theuring

**Affiliations:** a Institute of International Health, Charité – Universitätsmedizin Berlin, corporate member of Freie Universität Berlin and Humboldt Universität zu Berlin, Campus Virchow Klinikum, Augustenburger Platz 1, Berlin 13353, Germany; b Initial Reception Examination Center, Charité – Universitätsmedizin Berlin, corporate member of Freie Universität Berlin and Humboldt Universität zu Berlin, Campus Charité Mitte, Charitéplatz 1, Friedrich-Althoff Haus, Berlin 10117, Germany; c Institute of Public Health, Charité – Universitätsmedizin Berlin, corporate member of Freie Universität Berlin and Humboldt Universität zu Berlin, Luisenstraße 57, Berlin 10117, Germany; d Gender Health and Justice Research Unit, Faculty of Health Sciences, University of Cape Town, Falmouth Building, Anzio Road Observatory, Cape Town 7925, South Africa; e Medical Directorate, Charité – Universitätsmedizin Berlin, corporate member of Freie Universität Berlin and Humboldt Universität zu Berlin, Campus Charité Mitte, Charitéplatz 1, Friedrich-Althoff Haus, Berlin 10117, Germany

**Keywords:** Migration, Health policy, Refugee health, Health services research, Sexual and reproductive health

## Abstract

**Introduction:**

Germany is a major destination country for asylum-seekers, including from many countries where female genital mutilation/cutting (FGM/C) is practiced. Our study aimed to provide empirical data on FGM/C prevalence, associated health complaints and FGM/C-related perceptions among asylum-seeking women in Berlin.

**Methods:**

A cross-sectional study was conducted among asylum-seeking women in Berlin, Germany between October 2018 and September 2022. Adult women self-reporting FGM/C were interviewed using a questionnaire querying FGM/C type, physical complaints, and pain domains, and open-ended questions on women’s experiences regarding FGM/C.

**Results:**

In total, 15,167 women underwent the mandatory medical examination for asylum-seekers during the study period, 1.9% of which self-reported FGM/C. The prevalence reached 18.6% when restricting the population to FGM/C-practicing countries. Women living with FGM/C came predominantly from the African WHO region (61.9%). A total of 151 women consented to being interviewed. The majority (57.0%) self-allocated to FGM/C Type I/II. Menstrual pain was the most reported complaint (77.5%), followed by pain during defecation (29.1%). Most physical complaints were reported by slightly higher proportions among women with FGM/C type III. Women reported a median age of six years at FGM/C execution, which was performed largely by female, non-relatives (64.5%). The majority (90.1%) perceived FGM/C negatively while 7.9% had a neutral or positive attitude towards it due to its cultural significance.

**Conclusion:**

FGM/C is prevalent in host countries like Germany with a potentially associated physical and psychosocial health burden. Our findings emphasize the need for a holistic, culturally sensitive response to such health demands by healthcare systems.

## 1 Introduction

Female genital mutilation/cutting (FGM/C) is the non-medical injury to or removal of external female genitalia. (World Health Organization 2019) It encompasses a range of practices from partial or total removal of the clitoral glans and/or clitoral hood (WHO Type I) with inclusion of labia minora and/or majora (WHO Type II) to cutting and repositioning the labia minora and/or majora with subsequent narrowing of the vaginal opening (WHO Type III) as well as other harmful procedures such as piercing or cauterization of the genitalia (WHO Type IV). (World Health Organization 2019)

The practice of FGM/C is found all over the globe and has strong cultural roots. It is considered a rite of passage into womanhood in many cultures e.g. from Northeastern and Western Africa, and its continuation is fueled by social acceptance or even social desirability to preserve the women and their families’ good standing in society and avoid social exclusion. (Williams-Breault, 2018; Van Rossem et al., 2015) While FGM/C is entrenched in gender inequality, the perpetuation of certain gendered body norms, as well as patriarchal social norms in the societies where it is practiced, (Villani, 2023; Jacobson et al., 2018; Klein et al., 2018) in some cultures it is conversely perceived to increase female bonding, solidarity, and empowerment, in effect countering patriarchy and male dominance in those cultures. (Gruenbaum et al., 2023) Notwithstanding the motivation behind FGM/C, the international consensus – potentially deviating from the discourse in practicing countries – is that it represents a violation of multiple human rights including, but not limited to, the right to equality, non-discrimination based on sex, as well as children´s rights as it is predominantly conducted in minors. (Williams-Breault, 2018)

FGM/C is linked to a multitude of physical health risks. Short-term complications include bleeding and infections due to the often unsanitary conditions under which FGM/C is performed, aggravated by the lack of availability of medical care in some settings. (Klein et al., 2018) The procedure is conducted within medical facilities in some countries such as Egypt as a harm reduction strategy; (El-Gibaly et al., 2019) yet the medicalization of FGM/C is considered dangerous and unethical by some as it trivializes its harmful consequences. (Boddy, 2020) Long-term physical health risks can include development of cysts, painful urination, complications during childbirth, and possible infertility. (Klein et al., 2018) Sexual function can also be affected by FGM/C, being linked to diminished sexual pleasure, decreased lubrication and dyspareunia. (Shafaati Laleh et al., 2022) Furthermore, its impact extends to include long-term risks to mental wellbeing. (Adam, 2021) FGM/C has been shown to be associated with post-traumatic stress disorder (PTSD), depression, anxiety, somatization, and phobia, (Im et al., 2020; Buggio et al., 2019) to which additional mental health effects might be added particularly among refugee women due to the risk of physical, psychological or sexual trauma before, during and after flight. (Vromans et al., 2021) The health consequences as well as risk of mortality further constitute violations to the right to health and right to life respectively. (Khosla et al., 2017)

A recent meta-analysis of studies published between 2009–2022 reported an overall FGM/C prevalence in practicing countries of 36.9% among women aged between 15–49 years, whereas it reached 8.3% among girls aged between 0–15 years. (Farouki et al., 2022) Country-specific prevalence within the aforementioned period ranged from 0.3% in Uganda to 99.2% in Somalia for women, (Uganda Bureau of Statistics 2018; Uganda Bureau of Statistics 2020) and from 0.1% in Ghana to 72.7% in Mali for girls. (Ghana Statistical Service 2018; Institut National de la Statistique (INSTAT) 2018) According to the United Nations Children’s Fund (UNICEF), over 230 million girls and women worldwide are living with FGM/C, with the majority in the African continent followed by Asia and the Middle East, and most of which are subjected to FGM/C between the ages of six and twelve. (Klein et al., 2018; United Nations Children's Fund (UNICEF) 2019) Between 2019 and 2023, the number of girls who were at risk of FGM/C increased from 4.1 to 4.3 million according to the United Nations Population Fund (UNFPA) and is expected to increase to 4.6 million by 2030, even if current elimination efforts by different stakeholders including UNFPA and UNICEF are sustained. (United Nations Population Fund (UNFPA) 2023)

The presence of FGM/C in EU countries like Germany has increased in the last few years due to the influx of migrants from countries in which it is practiced. (En-Nosse et al., 2022) As of 2022, it has been estimated that 103,947 girls and women in Germany are living with FGM/C. (Terre des Femmes e.V., Weibliche Genitalverstümmelung in Deutschland: Dunkelzifferschätzung 2022 2022) However, empirical estimates of FGM/C prevalence are lacking, and reported prevalence in Germany is estimated based on its prevalence in migrants’ home countries, potentially resulting in inaccurate estimations due to possible ambiguities in migrants’ countries of origin or affiliation to countries in which regional differences exist. (En-Nosse et al., 2022; Terre des Femmes e.V., Weibliche Genitalverstümmelung in Deutschland: Dunkelzifferschätzung 2022 2022) Furthermore, there is limited understanding of the healthcare needs of women living with FGM/C within the context of flight regarding physical, psychosocial, sexual and reproductive health, taking into consideration their experiences and perceptions regarding the practice.

Acknowledging that Germany received the highest number of migrants among EU countries in recent years such as in 2022, (Eurostat. Migration and migrant population statistics 2024) the determination of FGM/C prevalence and a better understanding of their situation is essential for adequate healthcare provision to migrant women according to Germany’s commitment to protect and support women living with FGM/C as stated in the Istanbul convention; (Council of Europe 2014) thus, contributing to achieving the Sustainable Development Goal 5.3 regarding the elimination of harmful practices including FGM/C. To this end, the current study aims to report the prevalence of FGM/C among asylum-seeking women arriving in Berlin between October 2018 and September 2022, describe potentially FGM/C-associated health complaints, and explore perceptions and experiences linked with FGM/C.

## 2 Methods

### 2.1 Study design and setting

We conducted a descriptive cross-sectional study among women seeking asylum in the state of Berlin between October 2018 and September 2022 who underwent mandatory medical examination required for asylum-seekers under § 62(1) of the German Asylum Act. The examination is carried out after arrival and registration in the initial reception center, but before application for asylum. It is conducted in the medical facility located within the initial reception center, which was the only site in Berlin where this examination was performed. It primarily aims to detect infectious diseases, with particular emphasis on excluding tuberculosis. While the exact scope varies by federal state, the examination typically includes a medical history, a general physical examination for signs of infection, and vaccination if necessary. As tuberculosis screening requires radiological imaging, asylum-seeking women are specifically asked about their pregnancy status; however, no further gynecological examination is performed as it is not part of the mandatory medical examination. (Robert Koch Institute 2015)

### 2.2 Study procedure and data collection

All women who underwent the mandatory medical examination in our study period were asked if they had undergone FGM/C. In the case of underage girls, parents were asked about their FGM/C status. This information was used for prevalence calculation, but no other data was collected from this group. Women aged 18 years or older who reported having experienced FGM/C were invited to participate in our study. Individuals below 18 years of age were not invited to participate in the survey to avoid potential retraumatization related to questions on their perceptions and experiences with FGM/C. To ensure the cultural sensitivity, data were collected by female physicians with subject-matter knowledge on FGM/C, who had experience in working with asylum-seekers. A self-developed, semi-structured questionnaire was administered in easily understandable language with the help of qualified interpreters when needed. FGM/C status was ascertained via self-report by the participants. Physical confirmation of the self-reported FGM/C status was not conducted as the medical facility was not equipped to perform gynecological examinations.

The questionnaire included sociodemographic data such as date of birth, country of origin and educational level as a categorical variable (*No education, Primary school, Middle/high school or University*); and FGM/C-specific data such as age at procedure as a continuous variable, and background on the person carrying out FGM/C, including their sex and whether they were related to the participant. FGM/C type was determined in accordance with the approach used in Demographic and Health Surveys (DHS). (Yoder et al., 2004) Participants were asked to describe the FGM/C type, which was then classified according to the WHO classification (World Health Organization 2019). Due to the difficulty in differentiation between types I and II, both were combined in one category to create a final categorical variable as follows: *type I/II, type III* or *type unclear*. Further data were collected on different domains of somatic pain including anal, vaginal, and menstrual pain, as well as pain during urination and defecation. These domains were chosen to cover the entire spectrum of possible pain areas, also considering the potential culturally varying expression of physical pain, especially for types of pain that may be stigmatized in certain contexts. (Okolo et al., 2024) For each of the domains, women were asked whether they experienced pain or not, and if so, to indicate the respective pain severity on a visual analogue 0–10 scale. We categorized the scale as *0, 1–4, or ≥5* for analysis. Presence of hypermenorrhea, fecal and urinary incontinence, and regularity of the menstrual cycle were also recorded as binary variables. Women’s perceptions on FGM/C were assessed using open-ended questions. While some of the interviewers noted these as bullet points, others entered a verbatim transcription of the woman´s statement in the questionnaire.

Women reporting symptoms related to FGM/C were provided with information on available counseling services, specialized care centers, or, in cases of severe symptoms, were referred to specialized medical facilities.

### 2.3 Ethical considerations

The study was approved by the Ethics Committee of Charité – Universitätsmedizin Berlin (EA1/131/18) and conducted according to the principles of the Declaration of Helsinki regarding research on human subjects. All participants provided their informed consent before participation. They were also informed about the possibility to withdraw from the study at any point. All data related to FGM/C were pseudonymized and stored on a separate server, distinct from routine medical records. The data collected on FGM/C were not shared as part of the asylum process.

### 2.4 Data analysis

Two FGM/C prevalence values were calculated. For the first calculation, all screened women during the study period were included yielding a total sample of 15,167 women. A further calculation was conducted including only those countries of origin in which FGM/C is practiced according to nationally representative data between 2009 and 2022 (29 countries) (Farouki et al., 2022), yielding a subsample of 1447 women.

Quantitative data were descriptively reported as absolute and relative frequencies for categorical variables, whereas continuous data were reported as means and standard deviations (SD) or as medians and interquartile ranges according to their distribution. Quantitative data analysis was conducted using R (Version 4.3.1; R Foundation for Statistical Computing, Vienna, Austria).

Responses to the open-ended questions were analyzed using the abridged procedures for qualitative thematic analysis. The responses were coded inductively by two independent investigators, and codes were then combined and grouped into categories to define main emerging themes. Statements that had been transcribed verbatim during the interviews were chosen to illustrate the overall findings. Study results were then reported according to the Reporting of Observational Studies in Epidemiology (STROBE) statement (Supplement A).

## 3 Results

**Fig. 1:**
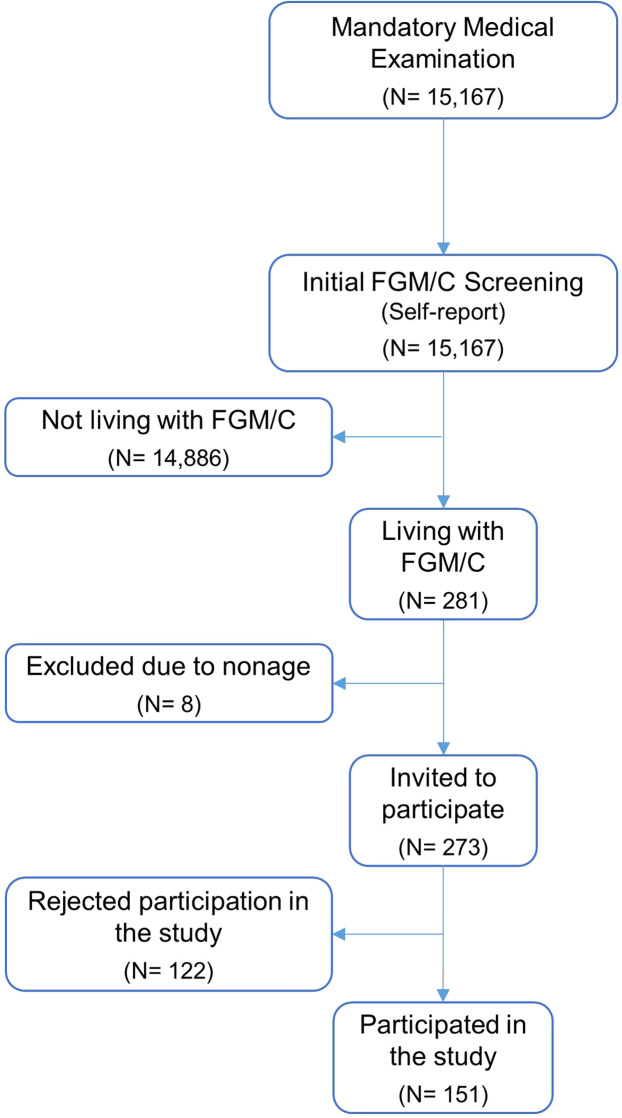
Flow diagram showing the recruitment of the study population.

Out of the total study population of 15,167 women, 14,886 reported not having experienced FGM/C, and 281 women stated they had (1.9%). Out of those who screened positive for FGM/C, eight were excluded as they were younger than 18 years (8/281; 2.8%), leaving 273 women who were invited to participate in the study. Further, 122 women rejected participation (122/281; 43.4%) yielding a final study population of 151 women living with FGM/C who were administered the questionnaire. (Fig. 1.) When restricting the study population to FGM/C-practicing countries (Farouki et al., 2022), the prevalence of FGM/C increased to 18.6% (269/1447). The restriction to these countries resulted in exclusion of 12 women living with FGM/C whose countries of origin were either unknown or lacked nationally representative data on FGM/C.

### 3.1 Participant characteristics

**Fig. 2:**
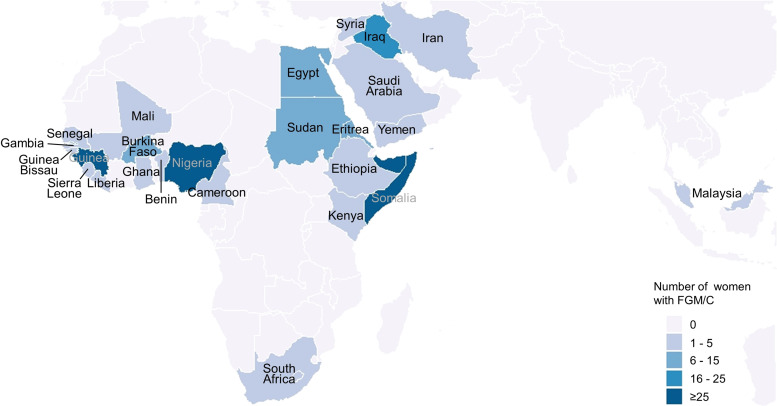
Countries of origin of women living with FGM/C – The map shows the countries from which women living with FGM/C come which are color-coded according to the respective number of women.

Table 1 shows the baseline characteristics of the study participants stratified by FGM/C status and participation in the study. The mean age (SD) of the study sample was 25.9 (15.9) years with women living with FGM/C having a higher mean age (SD) than those who were not (30.1 (10.6) vs. 25.8 (16.0)). Most women living with FGM/C came from the African WHO region (61.9%), primarily from Guinea (22.1%), followed by the Eastern Mediterranean WHO region (36.7%), primarily from Somalia (16.7%) (Fig. 2; Supplement Table 1). Women who did not experience FGM/C came primarily from the European WHO region (53.9%) No major differences were found regarding urban/rural divide and educational level between women living with FGM/C and those who were not.

**Table 1:** Baseline characteristics of screened women by FGM/C status and participation status.

		Living with FGM/C			Not living with FGM/C N = 14,886	Total N = 15,167
		Participants N = 151	Non-participants N = 130	Total N = 281		
Age	Mean (SD)	30.1 (8.9)	30.1 (12.3)	30.1 (10.6)	25.8 (16.0)	25.9 (15.9)
	Missing	0	2 (1.5%)	2 (0.7%)	80 (0.5%)	82 (0.5%)
WHO Region	African	99 (65.6%)	75 (57.7%)	174 (61.9%)	465 (3.1%)	639 (4.2%)
	Americas*	0 (0%)	0 (0%)	0 (0%)	201 (1.4%)	201 (1.3%)
	Eastern Mediterranean	51 (33.8%)	52 (40.0%)	103 (36.7%)	4358 (29.3%)	4461 (29.4%)
	European*	0 (0%)	0 (0%)	0 (0%)	7944 (53.4%)	7944 (52.4%)
	South-East Asia	0 (0%)	0 (0%)	0 (0%)	24 (0.2%)	24 (0.2%)
	Western Pacific	1 (0.7%)	0 (0%)	1 (0.4%)	1664 (11.2%)	1665 (11.0%)
	Unclassified	0 (0%)	0 (0%)	0 (0%)	133 (0.9%)	133 (0.9%)
	Missing	0 (0%)	3 (2.3%)	3 (1.1%)	97 (0.7%)	100 (0.7%)
Rural / Urban	Urban	89 (58.9%)	60 (46.2%)	149 (53.0%)	7300 (49.0%)	7449 (49.1%)
	Rural	58 (38.4%)	64 (49.2%)	122 (43.4%)	6431 (43.2%)	6553 (43.2%)
	Missing	4 (2.6%)	6 (4.6%)	10 (3.6%)	1155 (7.8%)	1165 (7.7%)
Level of Education	University	21 (13.9%)	14 (10.8%)	35 (12.5%)	1506 (10.1%)	1541 (10.2%)
	Middle/High School	51 (33.8%)	32 (24.6%)	83 (29.5%)	3512 (23.6%)	3595 (23.7%)
	Primary School	37 (24.5%)	45 (34.6%)	82 (29.2%)	3749 (25.2%)	3831 (25.3%)
	No Education	39 (25.8%)	32 (24.6%)	71 (25.3%)	4973 (33.4%)	5044 (33.3%)
	Missing	3 (2.0%)	7 (5.4%)	10 (3.6)	1146 (7.7%)	1156 (7.6%)

*The European / Americas regions include high-income countries. This could be due to some participants referring to the country where they travelled to Germany from and not necessarily their country of origin.

### 3.2 Physical complaints

Out of the five assessed pain domains, 57.6% of women reported experiencing pain in one or two domains, 24.5% experienced pain in three or more domains, and 15.2% did not report experiencing any pain. Table 2 shows the proportion of women reporting different types of pain and its severity. Across all pain domains, a higher proportion of participants reported experiencing menstrual pain (77.5%) compared to the other domains ranging from 11.9% reporting anal pain to 29.1% reporting pain during defecation. Regarding pain severity, vaginal pain and pain during defecation did not vary widely between participants with FGM/C types I/II and those with FGM/C type III. Almost a quarter of participants with FGM/C type III reported severe urinary pain compared to those with FGM/C type I/II (24.2 vs. 9.3%). When asked about pain severity, the proportion of participants reporting moderate or severe menstrual pain (93.4%) was higher than those who reported experiencing menstrual pain at all (77.5%). Such inconsistency was not found in the other pain domains.

**Table 2:** Physical complaints of women living with FGM/C by FGM/C type.

Domain		Age Mean (SD)	Category	FGM/C Type			Total N = 151
				Type I/II N = 86	Type III N = 33	Unclear N = 32	
Anal pain	Dichotomous	30.5 (9.2)	No	74 (86.0%)	26 (78.8%)	30 (93.8%)	130 (86.1%)
		27.9 (6.4)	Yes	12 (14.0%)	5 (15.2%)	1 (3.1%)	18 (11.9%)
			Missing	0	2 (6.0%)	1 (3.1%)	3 (2.0%)
	Severity	30.4 (9.2)	0	70 (81.4%)	26 (78.8%)	30 (93.8%)	126 (83.4%)
		29.1 (5.4)	1–4	5 (5.8%)	4 (12.1%)	0	9 (6.0%)
		26.8 (7.4)	≥5	7 (8.1%)	1 (3.0%)	1 (3.1%)	9 (6.0%)
			Missing	4 (4.7%)	2 (6.1%)	1 (3.1%)	7 (4.6%)
Vaginal pain	Dichotomous	30.5 (9.1)	No	70 (81.4%)	25 (75.8%)	24 (75.0%)	119 (78.8%)
		28.7 (8.1)	Yes	16 (18.6%)	7 (21.2%)	7 (21.9%)	30 (19.9%)
			Missing	0	1 (3.0%)	1 (3.1%)	2 (1.3%)
	Severity	30.4 (9.1)	0	66 (76.7%)	26 (78.8%)	24 (75.0%)	116 (76.8%)
		30.0 (7.8)	1–4	3 (3.5%)	2 (6.1%)	3 (9.4%)	8 (5.3%)
		28.1 (8.5)	≥5	13 (15.1%)	4 (12.1%)	4 (12.5%)	21 (13.9%)
			Missing	4 (4.7%)	1 (3.0%)	1 (3.1%)	6 (4.0%)
Menstrual pain	Dichotomous	34.1 (10.6)	No	23 (26.7%)	4 (12.1%)	5 (15.6%)	32 (21.2%)
		29.1 (8.1)	Yes	63 (73.3%)	28 (84.9%)	26 (81.2%)	117 (77.5%)
			Missing	0	1 (3.0%)	1 (3.1%)	2 (1.3%)
	Severity	23.3 (4.4)	0	1 (1.2%)	2 (6.1%)	1 (3.1%)	4 (2.6%)
		31.1 (9.6)	1–4	39 (45.3%)	12 (36.3%)	12 (37.5%)	63 (41.7%)
		29.5 (8.4)	≥5	44 (51.2%)	17 (51.5%)	17 (53.2%)	78 (51.7%)
			Missing	2 (2.3%)	2 (6.1%)	2 (6.2%)	6 (4.0%)
Urinary pain	Dichotomous	30.5 (9.3)	No	64 (74.4%)	23 (69.7%)	26 (81.3%)	113 (74.8%)
		29.1 (7.6)	Yes	22 (25.6%)	10 (30.3%)	5 (15.6%)	37 (24.5%)
			Missing	0	0	1 (3.1%)	1 (0.7%)
	Severity	30.6 (9.5)	0	62 (72.1%)	18 (54.5%)	24 (75.0%)	104 (68.9%)
		32.6 (8.1)	1–4	13 (15.1%)	2 (6.1%)	1 (3.1%)	16 (10.6%)
		26.5 (6.2)	≥5	8 (9.3%)	8 (24.2%)	4 (12.5%)	20 (13.2%)
			Missing	3 (3.5%)	5 (15.2%)	3 (9.4%)	11 (7.3%)
Defecation pain	Dichotomous	30.8 (9.4)	No	58 (67.4%)	23 (69.7%)	25 (78.1%)	106 (70.2%)
		28.6 (7.3)	Yes	28 (32.6%)	10 (30.3%)	6 (18.8%)	44 (29.1%)
			Missing	0	0	1 (3.1%)	1 (0.7%)
	Severity*	30.8 (9.5)	0	56 (65.1%)	21 (63.6%)	25 (78.1%)	102 (67.6%)
		27.6 (6.8)	1–4	13 (15.1%)	6 (18.2%)	2 (6.2%)	21 (13.9%)
		29.5 (7.7)	≥5	15 (17.4%)	4 (12.1%)	4 (12.5%)	23 (15.2%)
			Missing	2 (2.3%)	2 (6.1%)	1 (3.1%)	5 (3.3%)
Urinary Incontinence		30.1 (9.1)	No	71 (82.6%)	28 (84.8%)	29 (90.6%)	128 (84.8%)
		30.5 (7.9)	Yes	15 (17.4%)	5 (15.2%)	2 (6.3%)	22 (14.5%)
			Missing	0	0	1 (3.1%)	1 (0.7%)
Fecal Incontinence*		30.3 (9.0)	No	81 (94.2%)	32 (97.0%)	30 (93.8%)	143 (94.7%)
		27.3 (5.7)	Yes	3 (3.5%)	1 (3.0%)	0	4 (2.6%)
			Missing	2 (2.3%)	0	2 (6.2%)	4 (2.6%)
Hypermenorrhea		30.2 (9.1)	No	72 (83.7%)	24 (72.7%)	25 (78.1%)	121 (80.1%)
		30.0 (8.2)	Yes	14 (16.3%)	9 (27.3%)	6 (18.8%)	29 (19.2%)
			Missing	0	0	1 (3.1%)	1 (0.7%)
Cycle Length		31.5 (9.4)	Regular	47 (54.6%)	21 (63.6%)	24 (75.0%)	92 (60.9%)
		28.2 (7.8)	Irregular	33 (38.4%)	12 (36.4%)	7 (21.9%)	52 (34.5%)
			Unclear	6 (7.0%)	0	1 (3.1%)	7 (4.6%)

*Some percentages do not add exactly up to 100% due to rounding.

The prevalence of urinary and fecal incontinence did not differ much between different types of FGM/C reaching 14.6% and 2.6%, respectively. On the other hand, the proportion of hypermenorrhea was higher in women with type III FGM/C compared to those with type I or II (27.3%vs. 16.3%). Cycle length was reported to be irregular in a comparable proportion of women with type III FGM/C (38.4%) and type I or II FGM/C (36.4%).

### 3.3 Experiences and perceptions of FGM/C

Most of the women experienced FGM/C at primary school age, performed through a female, mostly non-related person (Table 3).

**Table 3:** FGM/C-related characteristics.

Variable	Category	Total N = 151
Age at FGM/C	Median (IQR)	6 (3, 9)
	Missing	22 (14.6%)
Person conducting FGM/C	Female relative	13 (8.6%)
	Female non-relative	98 (64.9%)
	Male relative	1 (0.7%)
	Male non-relative	9 (6.0%)
	Unclear	30 (19.9%)
FGM/C Type	Type I/II	86 (57.0%)
	Type III	33 (21.9%)
	Unclear	31 (20.5%)
	Missing	1 (0.7%)

*Some participants were not able to mention clearly the person who performed FGM/C due to, among other reasons, being very young at the time or to avoid burdening family members who may have been involved.

In their responses to the open-ended questions, participating women emphasized physical complaints, especially experiencing pain, for example during urination: “*The circumcision was very bad and painful. I couldn't go to the toilet properly for four months and couldn't urinate without pain.*” Moreover, 21 women reported further complaints during sexual intercourse such as dyspareunia and lack of sexual pleasure: “*Sex is very painful. There's no pleasure in it. Every touch hurts.*” The negative impact on sexuality caused some to refrain from living it out altogether, expressed as not having “*desire for men*” or “*the courage to get married*”.

Women also reported further long-term psychosocial negative consequences, partly due to the “*traumatizing*” pain experienced during the procedure. An 18-year-old Guinean woman reported having nightmares due to her experience with FGM/C as follows: “*I still cannot forget the pain from that day. I still have nightmares about it. I was forced to do it back then; it was violence, very bad.*” Others described experiencing FGM/C as having destroyed their lives preventing them from experiencing joy anymore. The negative effect of FGM/C on women’s self-image was also frequently mentioned by participants as described by a 41-year-old woman from Burkina Faso: “*I feel like I’m different when I’m with other women who are not circumcised.”* Others described themselves as *“excluded”* and *“incomplete”.* Further, the psychosocial aspect of FGM/C-related obstetric consequences was reflected on by a 23-year-old Guinean woman as follows: “*Circumcision has destroyed the dream of becoming a mother in the normal way for me and every other woman.*”. This is in line with the experiences of other women who had to undergo Caesarean section more than once due to FGM/C.

The responses delivered further insights into perceptions of women on the FGM/C practice which were overwhelmingly negative, with many describing the practice as a form of physical violence, “*evil*”, “*inhumane*”, “*a crime*”, or “*an injustice*”. It was reported to be a deeply entrenched tradition that is associated with physical and social coercion. One woman from Burkina Faso, aged 29, highlighted the socially coercive aspect of FGM/C on her family despite being opposed to it: *“My parents could not do anything, they didn't want [FGM/C], but my parents-in-law forced me and my parents to do it.”* Such coercion is driven by fear of social exclusion as reported by a 61-year-old Iranian woman: “*Many people believed that all women had to be circumcised, otherwise no one was allowed to eat or drink with her.*” Many women linked FGM/C to societal control of female sexuality, emphasizing the patriarchal societal norms driving this practice. They reported that the practice is culturally perceived to protect women from promiscuity or infidelity as articulated by a 35-year-old Ethiopian woman: “*Most women are afraid of circumcision, but most people find it good, as it protects [women] from sexual misbehavior.”* However, women opposed this view as expressed by a 37-year-old Nigerian woman: “*They do it so that women obey men. They think that women who are not circumcised are not faithful and do whatever they want. But it's not because of circumcision. If women are treated badly, harassed, and neglected, then they leave.*” Another form of control on women’s bodies was described by a Sudanese woman who said that men ask midwives to sew up women’s vulvas again after childbirth. Conversely, only 12 women had neutral and positive perceptions on FGM/C. Neutral perceptions were attributed to lack of pain during the procedure, lack of current complaints and that it is a cultural norm. A woman who perceived FGM/C positively vocalized this as “*I think it is good, but I do not have much information*”.

The perceptions on discontinuation of the practice varied along generational, socioeconomic, and geographic lines. FGM/C was reported to be increasingly rejected among younger generations and educated women, especially those living in larger cities compared to in rural areas. Furthermore, some women mentioned governmental efforts to stop FGM/C such as its criminalization which had limited success: “*Before I fled, the father of my 3-year-old daughter wanted to circumcise her according to tradition. It [FGM/C] is legally prohibited in my country, but it continues in secret.*” Others highlighted the role of awareness raising by individuals or organizations in stopping FGM/C. A 45-year-old woman from Burkina Faso recalled: “*I think this is very bad, so I tried to make the other women aware of it. I complained about it to the police with these women.*” Some women linked their perception to the type of circumcision, being explicitly against type III FGM/C as opposed to milder forms of FGM/C as a 42-year-old Sudanese woman reported: “*My own circumcision is fine, “sunna” [milder FGM/C forms] it doesn't hurt, it's not dangerous. But I am against pharaonic circumcision [type III FGM/C], women suffer greatly.*” The rejection of circumcision of daughters as mentioned by many women further emphasized their negative perceptions on the practice with some highlighting the need to protect their daughters from it as the reason they had fled their countries of origin. Conversely, two women intended to have their daughters circumcised; however, one of them emphasized: “*I do not know if [FGM/C] is right or not. If I have a daughter, I would circumcise her, but I will not force her.”*

## 4 Discussion

This study is among the first to report empirical data about FGM/C prevalence and physical complaints among asylum-seeking women in Germany. Between October 2018 and September 2022, 1.9% of incoming asylum-seeking women stated that they were living with FGM/C. Considering only women from FGM/C practicing countries (Farouki et al., 2022), the respective subsample showed a prevalence of 18.6%, mostly reporting FGM/C type I or II. Participating women reported considerable pain burden, especially pain during menstruation, defecation and urination. They also reported psychosocial as well as sexual complaints directly related to living with FGM/C. Women’s perceptions of the FGM/C practice were overwhelmingly negative describing it as a form of violence, injustice, and societal control of women and their sexuality. The majority of women emphasized their opposition to the continuation of the practice citing its harmful effects on their physical and psychosocial well-being.

Estimation of FGM/C prevalence and comparing our estimate with previously published studies proved challenging. Self-reporting of FGM/C status is subject to underreporting, possibly due to fear of stigmatization, awareness of its illegality, traumatic experience, or social desirability. (O'Neill and Pallitto, 2021; Jackson et al., 2003) The latter could have been particularly pronounced in our study due to its setting, as some women could have feared negative implications for the asylum process. Women could also be unaware of having experienced minimal forms of FGM/C especially when performed at a young age. (Klouman et al., 2005) Comparing our results to those of previous studies conducted in Germany and other European countries is difficult and of limited significance, as the proportion of women living with FGM/C in these studies varied widely due to different sample sizes, varying estimation periods, and the difference of population characteristics regarding their countries of origin, and whether they were migrants or asylum-seekers. (Kawous et al., 2020; Zinka et al., 2018; Koschollek et al., 2020; Loucas et al., 2018; Fawcett and Kernohan, 2018; Ford et al., 2018) Available numbers of women and girls potentially living with FGM/C in Germany are calculated through the extrapolation of FGM/C prevalence in home countries to the corresponding migrant population in Germany. (Terre des Femmes e.V., Weibliche Genitalverstümmelung in Deutschland: Dunkelzifferschätzung 2022 2022) Using the estimated number of women and girls living with FGM/C in the state of Berlin in proportion to the total number of registered migrant women and girls yielded an estimated prevalence of 33.6% in 2022. (Terre des Femmes e.V., Weibliche Genitalverstümmelung in Deutschland: Dunkelzifferschätzung 2022 2022) However, using FGM/C prevalence in migrants' home countries to extrapolate prevalence among migrant populations is prone to error, as it neglects the notion of immigrant selectivity, i.e., individuals who emigrate from a country differing systematically from those who stay in their country of origin regarding certain characteristics such as educational attainment. (Spörlein et al., 2020) This is especially relevant in the context of FGM/C, as the higher educational level of mothers was reported to be associated with a lower risk of their daughters undergoing FGM/C. (Ackah et al., 2022)

### 4.1 Physical health manifestations

Most study participants reported experiencing one or more types of pain in addition to further physical complaints such as fecal or urinary incontinence and hypermenorrhea, with menstrual pain being the most reported type of pain. Women living with FGM/C, especially with type III, may experience more severe menstrual pain due to vaginal obstruction and subsequent hindrance of blood flow. (Rushwan, 2013) Increased infections caused by poor hygiene conditions and menstrual hygiene particularly during flight could contribute to a high proportion of women reporting severe menstrual pain. (Gammoh et al., 2024) The plausible role of FGM/C in aggravating menstrual pain is supported by the higher proportion of women with FGM/C type III reporting it compared to those with types I or II. Similarly, injury to the urinary canal or its obstruction caused by FGM/C and subsequent infections could lead to painful urination. (Rushwan, 2013) Women living with FGM/C are reported to have a higher risk of perineal tears during childbirth due to scar tissue formation, also with a higher risk in women with FGM/C type III. (Wuest et al., 2009) These have been reported to result in anal pain, as well as fecal and urinary incontinence. (Laine et al., 2011; Macarthur and Macarthur, 2004; Bols et al., 2010) Many women in our study also reported sexual health manifestations due to FGM/C, including dyspareunia and lack of sexual pleasure. Impaired sexual functioning in women affected by FGM/C has been shown to be linked to lower sexual desire, arousal, and lubrication. (Shafaati Laleh et al., 2022) This was attributed to the impaired sexual response due to excision of clitoral glans and vascular tissue removal which is responsible for lubrication. (Berg et al., 2010; Graziottin et al., 2023) Moreover, loss of elasticity due to formation of scar tissue and its tearing during intercourse are reported to cause dyspareunia. (Bazzoun et al., 2021) In alignment with our study results, sexual dysfunction could prevent women from exploring their sexuality, relationships, or marriage due to fear of pain and lack of desire. (Jordal et al., 2022; Abdulcadir et al., 2022) Previous studies reported that lack of sexual desire caused by FGM/C prompted women to tolerate sex with their partners for fear of rejection, caused them feelings of guilt and shame due to perceived inability to satisfy their partners, and even subjected them to forced marital sex by their partners. (Jordal et al., 2022; Esho et al., 2017) Due to the complex nature of sexuality and the contribution of psychological and sociocultural factors to its perception, (Johnson-Agbakwu and Warren, 2017) discussing sexual dysfunction merely as a function of FGM/C-induced physiological changes is reductive, especially in the migration context. The switch from a culturally favorable discourse about FGM/C in home countries to a pathologizing Western discourse could further alter women’s body image and self-esteem, thereby negatively affecting sexual function even in women who are able to experience sexual desire. (Jordal et al., 2022; Connor et al., 2016; Johnsdotter, 2018)

### 4.2 Psychosocial consequences

Understanding the psychosocial consequences of FGM/C requires a life-course approach considering cumulative traumata and the changing social context of migrant women through different life phases. (Im et al., 2020) In our study, some women described the vivid memories of circumcision and its lasting consequences. This could be due to experiencing severe pain, fear, and helplessness during circumcision, and might leave them vulnerable to PTSD at a rate comparable to that of early childhood abuse survivors. (Behrendt and Moritz, 2005) Feeling forsaken by their parents and the consequent damage to trust in them can further aggravate psychosocial consequences for FGM/C-experiencing children. (Parikh et al., 2020) Consequently, this poly-victimization of asylum-seeking women living with FGM/C in different life phases is associated with a high burden of mental ill-health. (Im et al., 2020; Buggio et al., 2019; Vromans et al., 2021) Encounters with healthcare systems in host countries that uphold stigmatizing views of FGM/C can perpetuate preexisting feelings of inferiority and incompleteness, as reported in our study, and contribute to mental ill-health. (Lever et al., 2019)

Despite how closely interlinked the physical and psychosocial consequences of FGM/C are, the interrelation between both aspects and its collective influence on the wellbeing of women living with FGM/C are still understudied. (Tammary and Manasi, 2023) In our study, participants mentioned physical and social coercion as an important aspect shaping their negative perception of FGM/C, as reflected by the assertion that they would not force their daughters to be circumcised even if they are not against FGM/C per se. The physical pain endured during immobilization and circumcision was described as traumatizing by study participants and have been found to amount to child abuse. (Zayed and Ali, 2012) Despite FGM/C being considered as an empowering counter-patriarchal practice in some cultures, (Gruenbaum et al., 2023) it was still perceived by women as a form of societal and male control over their bodies in our study.

Our results regarding perceptions on discontinuation of FGM/C reflect the multipronged nature of the approach required to sustainably prevent FGM/C. Such approach encompasses the regulatory aspect comprising criminalization of the practice as well as the social-cultural aspect emphasizing education and raising awareness on its detrimental physical and psychosocial consequences. (Sabi Boun et al., 2023) Increased urbanization and improvement of women’s socioeconomic conditions through education have also been reported as factors that support discontinuation of the practice, which was also shown in our study. (Dalal et al., 2010) This could be attributed to increased female empowerment and prioritizing individual autonomy and rights rather than perceived sociocultural obligations and norms. (Ortensi and Farina, 2014) The varying socioeconomic landscape of countries of origin represented in our sample could partly explain the wide range of perceptions of participating women.

Psychosocial consequences could be aggravated by FGM/C prevention strategies like criminalization in host countries, rendering them counterproductive and detrimental to migrant women’s psychosocial well-being. In alignment with our results, the criminalization of FGM/C in EU countries including Germany could cause the practice to continue in secret due to its deep-rooted cultural significance. (Thabet and Thabet, 2003; Barrett et al., 2020) Furthermore, consequent safeguarding practices, such as mandatory engagement of child protective services and potential child removal, portray women living with FGM/C as potential perpetrators who endanger their daughters. (Costello, 2015; González-Timoneda et al., 2021; Carver et al., 2024) Therefore, successful prevention of FGM/C requires a decolonial approach wherein community members including healthcare professionals from similar cultural backgrounds play a pivotal role in challenging social norms within migrant communities. Community-based interventions are time- and resource-intensive; however, they are postulated to bring about tangible and sustainable change due to their cultural sensitivity. (Barrett et al., 2020)

### 4.3 Implications for the German healthcare system

Almost 300 asylum-seeking women with FGM/C newly migrated to Berlin within four years, and cumulative numbers over time will continue to rise. Extrapolating the prevalence estimate of 1.9% to the total number of asylum-seeking women in Berlin yields a considerable number of women with complex healthcare needs. As such needs could manifest clinically as non-specific symptoms, healthcare professionals should consider FGM/C as a potential cause of such symptoms when treating women from FGM/C-practicing countries. (Kusi et al., 2025) Providing high-quality healthcare services for women living with FGM/C necessitates the understanding of such needs considering the intersectional stigma of migration, FGM/C, and possibly mental ill-health, and how these influence their already restricted access to healthcare services. This entails shifting from the prevalent pathologizing discourse on FGM/C in all aspects of healthcare provision without trivializing its adverse effects.

It has been established that in host countries including Germany, there is a myriad of barriers to high-quality healthcare for women living with FGM/C. (Schouler-Ocak et al., 2022) These include lack of knowledge among healthcare professionals about diagnosis and management of FGM/C; (Lurie et al., 2020; En-Nosse et al., 2023) language barriers and subsequent exclusion from decision making; (Evans et al., 2019) and experiences of racism. (Nationaler Diskriminierungs- and Rassismusmonitor (NaDiRa) 2023) Hence, high-quality care entails respectful communication and cultural sensitivity, through the inclusion of interpreters and cultural mediators when necessary. (Costello, 2015; Evans et al., 2019) Our study highlights further communication-related aspects regarding FGM/C. The discrepancy in menstrual pain reporting in our study suggests that health issues that may be considered normal or stigmatizing are potentially underreported; accordingly, they should be queried in various ways to uncover potential inconsistencies. Sexual and reproductive health-related concerns are surrounded by stigma and shame in some cultures; (Pérez-Sánchez et al., 2024) however, some participants in our study voluntarily addressed their own sexuality with regards to FGM/C, despite not being directly asked about it. This suggests that potentially medically or socially significant stigma-laden topics should be addressed taking into account a sensitive approach with trained personnel, preferably by individuals from a similar cultural background. (Ilami and Winter, 2021)

The interconnectedness of physical and psychosocial impacts of FGM/C necessitates the adoption of a holistic approach that provides healthcare services in a safe atmosphere, wherein interventions are considered beyond their potential biomedical gain. For example, seeking clitoral reconstruction surgery may not be restricted to restoring sexual function or reducing physical pain, but rather for other non-medical motives such as regaining power over one’s body or to have genitalia that are considered aesthetically “normal” within the new context. (Jordal et al., 2021) Furthermore, the current pathologizing discourse on sexual function can be undermined by providing information in a non-stigmatizing manner and fostering the role of intimate relationships with partners in achieving a satisfying sexual life. (Rushwan, 2013; Villani, 2023) Finally, aside from the potential psychotherapeutic need, psychosocial support should traverse all other aspects of healthcare provision to help navigate topics encountered within the new context such as body image as well as racism and xenophobia.

### 4.4 Strengths and limitations

The current study is one of the first empirical studies to report FGM/C prevalence and potentially related health outcomes among asylum-seeking women in Germany. Including all women regardless of their country of origin prevented the over- or underrepresentation of high-prevalence countries and subsequently prevented biased overall prevalence estimates. However, the results should be interpreted with caution as the study population is not representative for the entire population of asylum-seeking women in Berlin, due to potential difference in its composition over time with respect to countries of origin and due to potential relocation of women after arrival in Berlin. (Bundestag, 2016) This is especially relevant in light of the modest response rate in our study. However, the latter is to be expected, considering the recruitment setting and the vulnerability of the study population. As another limitation, the comparability of our results is limited by the lack of data from women who did not experience FGM/C. Comparability is further limited by the inclusion of asylum-seeking women only without migrant women with other types of residency status. Ascertainment of FGM/C status was done using self-report and was not confirmed via medical examination, rendering it vulnerable to recall or desirability bias and to misclassification with regards to FGM/C type. The frequency of experiencing physical pain, which differs across the different domains, was not considered. Finally, the target population was not involved in the planning of the research.

## 5 Conclusion

The empirical FGM/C prevalence estimate of our study points to a considerable number of FGM/C-affected women living in Berlin with complex physical and psychosocial health needs. However, similar studies including women who did not experience FGM/C are required to examine to what extent such physical and psychosocial needs are linked to FGM/C or to other causes, potentially related to flight and migration. In order to fulfill its obligations under the Istanbul Convention, the German healthcare system has to provide adequate healthcare to women affected by FGM/C. A holistic culturally sensitive approach mirroring the multifaceted nature of FGM/C and putting migrant women’s needs at the forefront of healthcare provision within their new social context is necessary. This requires collaborative development of care concepts with the involvement of stakeholders from the majority and minority worlds, to inform training of healthcare professionals, healthcare service design, further research, and prevention efforts that are acceptable and sustainable.

## Funding

This study was conducted within the Grand Challenges Initiative on Global Health - Exploration Project “MigraH”, funded under the Excellence Strategy of the Federal Government and the Länder by the Berlin University Alliance (BUA). The BUA had no input into the conduct and analysis of the study.

## Competing interests

The authors declare that they have no competing interests.

## Ethics approval

This study involves human participants. The study was approved by the Ethics Committee of Charité – Universitätsmedizin Berlin (EA1/131/18) and conducted according to the principles of the Declaration of Helsinki regarding research on human subjects. All participants provided their informed consent before participation. They were also informed about the possibility to withdraw from the study at any point. All data related to FGM/C were pseudonymized and stored on a separate server, distinct from routine medical records. The data collected on FGM/C were not shared as part of the asylum process.

## Data availability statement

To protect the vulnerable target group of the study, data is not publicly deposited. Data will however be made accessible upon reasonable request to the corresponding author.

## CRediT authorship contribution statement

**M.H. Barghouth:** Writing – review & editing, Writing – original draft, Visualization, Formal analysis. **E. Kusi:** Writing – original draft, Methodology, Investigation, Data curation. **A. Mueller:** Writing – review & editing, Formal analysis. **M. Kern:** Writing – review & editing, Formal analysis. **N. Bethke:** Writing – review & editing, Investigation, Data curation. **J. Seybold:** Writing – review & editing, Supervision, Project administration, Methodology, Investigation, Funding acquisition, Data curation, Conceptualization. **S. Theuring:** Writing – review & editing, Writing – original draft, Supervision, Project administration, Funding acquisition, Formal analysis.

## Declaration of competing interest

The authors declare that they have no known competing financial interests or personal relationships that could have appeared to influence the work reported in this paper.

## Appendix Supplementary materials

Image, application 1
